# Gut and bladder fermentation syndromes: a narrative review

**DOI:** 10.1186/s12916-023-03241-7

**Published:** 2024-01-22

**Authors:** Kenichi Tamama, Katherine M. Kruckenberg, Andrea F. DiMartini

**Affiliations:** 1 Clinical Laboratories, University of Pittsburgh Medical Center, Pittsburgh, PA USA; 2 Department of Pathology, University of Pittsburgh School of Medicine, Pittsburgh, PA USA; 3 McGowan Institute for Regenerative Medicine, University of Pittsburgh, 3477 Euler Way, UPMC Presbyterian Clinical Laboratory Building, Pittsburgh, PA 15213 USA; 4 Department of Psychiatry, University of California San Diego School of Medicine, San Diego, CA USA; 5 Departments of Psychiatry and Surgery, University of Pittsburgh School of Medicine, Pittsburgh, PA USA

**Keywords:** Auto-brewery syndrome, Fermentation, Candidiasis, Dysbiosis, Alcohol abstinence, Gut fermentation syndrome, Bladder fermentation syndrome

## Abstract

We recently reported the first clinical case of bladder fermentation syndrome (BFS) or urinary auto-brewery syndrome, which caused the patient to fail abstinence monitoring. In BFS, ethanol is generated by Crabtree-positive fermenting yeast *Candida glabrata* in a patient with poorly controlled diabetes. One crucial characteristic of BFS is the absence of alcoholic intoxication, as the bladder lumen contains transitional epithelium with low ethanol permeability. In contrast, patients with gut fermentation syndrome (GFS) or auto-brewery syndrome can spontaneously develop symptoms of ethanol intoxication even without any alcohol ingestion because of alcoholic fermentation in the gut lumen. In abstinence monitoring, a constellation of laboratory findings with positive urinary glucose and ethanol, negative ethanol metabolites, and the presence of yeast in urinalysis should raise suspicion for BFS, whereas endogenous ethanol production needs to be shown by a carbohydrate challenge test for GFS diagnosis. GFS patients will also likely fail abstinence monitoring because of the positive ethanol blood testing. BFS and GFS are treated by yeast eradication of fermenting microorganisms with antifungals (or antibiotics for bacterial GFS cases) and modification of underlying conditions (diabetes for BFS and gut dysbiosis for GFS). The under-recognition of these rare medical conditions has led to not only harm but also adverse legal consequences for patients, such as driving under the influence (DUI). GFS patients may be at risk of various alcohol-related diseases.

## Background

Ethanol fermentation by fermenting yeast is a well-known process. Similarly, yeast exists within the body as pathogens and commensal flora. Ethanol fermentation can occur within the gut lumen, causing spontaneous ethanol intoxication (gut fermentation syndrome or GFS). Ethanol fermentation within the oral cavity was also reported recently [1]. In 2020, we published the first experimentally-proven case of ethanol fermentation in the bladder (bladder fermentation syndrome or BFS), in which ethanol is generated by fermenting yeast *Candida glabrata* (*C. glabrata*) in the urinary bladder of a patient with poorly controlled diabetes female [2]. The patient sought a liver transplant for cirrhosis secondary to non-alcoholic steatohepatitis (NASH) (or metabolic dysfunction-associated steatohepatitis (MASH) under the new classification [3]), but it was misdiagnosed as alcohol-associated liver disease (ALD) due to multiple positive urine ethanol screens. As a result, she was deactivated from the waitlist despite the adamant denial of any alcohol consumption on her part.

Ethanol production by fermenting yeast and bacteria was reported in a urine specimen in vitro, as summarized by Gruszecki et al. [4], especially if the specimen tube is left outside the refrigerator. One forensic report described in vitro ethanol production by fermentation in the urine specimen of diabetic rape victims. The victims were falsely suspected of being intoxicated at the time of sexual assault because of high ethanol levels in their urine specimens [5]. In another forensic report, the authors retrospectively speculated that fermentation began in the bladder of a patient before death [4]. Integrating these previous cases, it is reasonable to conclude that fermentation occurs in the bladder where a patient is infected with or colonized by fermenting yeast with access to sugar. Nevertheless, this hypothesis was possibly overlooked or taken for granted until we conducted the first proof-of-principle experiment [2].

In this narrative review article, we overview BFS and further discuss (1) the pathophysiology, including the causative microorganism, Crabtree effect of yeast metabolism, key characteristics of urinary bladder epithelium, and intraluminal oxygen pressure of hollow organs; (2) the diagnostic tests, treatment, and medical and forensic significance of BFS; and (3) a comparative discussion of BFS with GFS.

## Pathophysiology of BFS

### Funguria (candiduria)

The presence of yeast in urine is called funguria or candiduria, as urinary yeast is almost always *Candida* species. Funguria reflects various conditions, ranging from yeast colonization in the urinary bladder to disseminated candidiasis [6], and is relatively common among hospitalized elderly inpatients. *C. albicans* is a predominant organism causing funguria, accounting for 50–70% of *Candida* isolates, whereas *C. glabrata* and *C. tropicalis* are the second and third dominant *Candida* isolates from funguria specimens [7].

Asymptomatic funguria represents bladder colonization with *Candida* spp. Funguria can also be part of systemic fungal infections such as urosepsis, and ethanol production by fermenting yeast has been noted in these cases [8, 9]. Even though there are some similar features (e.g., funguria, ethanol production in urine) shared between these cases and BFS, these cases should not be confused with BFS in our opinion.

### Crabtree effect

**Fig. 1:**
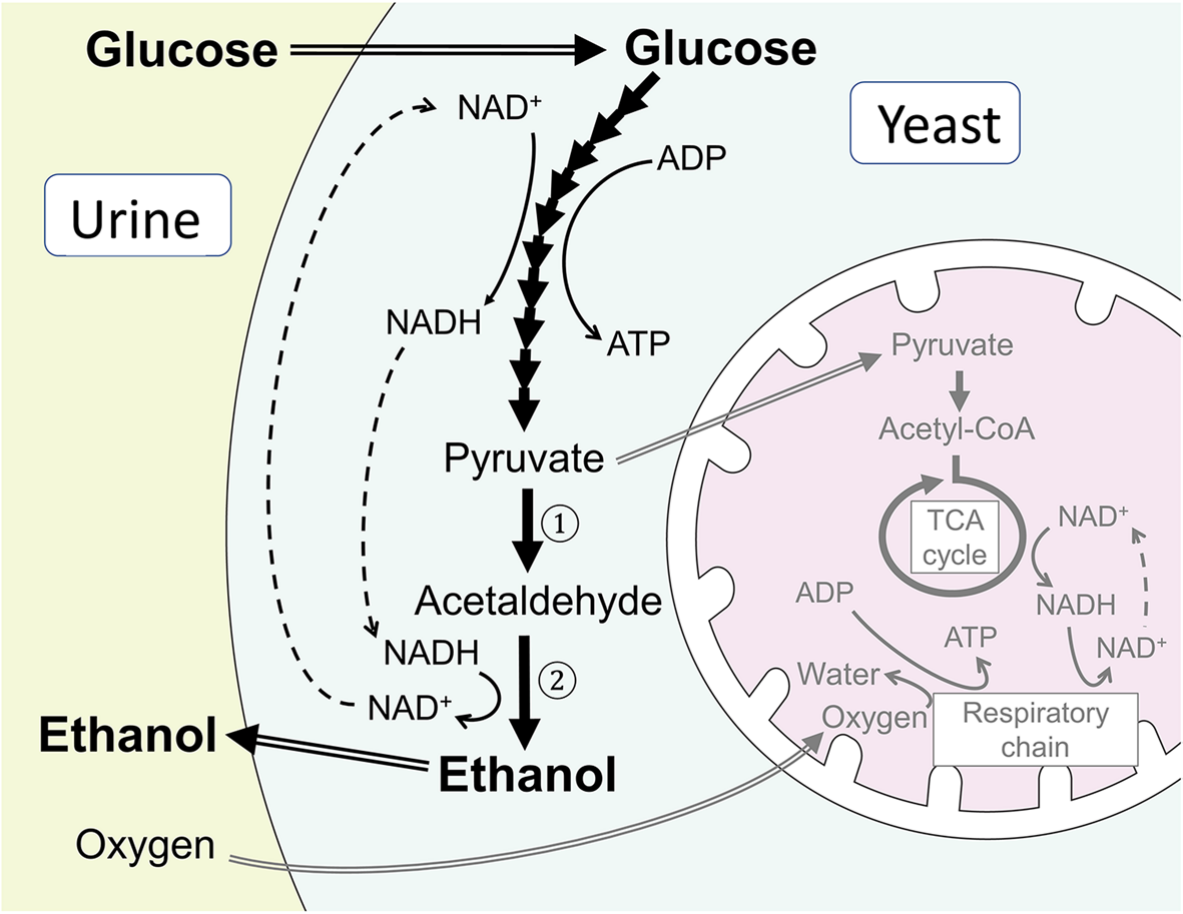
Simplified biochemical pathways illustrating glycolysis, alcoholic fermentation, and the TCA cycle in Crabtree-positive yeast. In Crabtree-positive yeast such as *Candida glabrata*, glucose is preferentially metabolized through glycolysis and alcoholic fermentation to ethanol in the cytosol without utilizing oxygen in the TCA cycle in mitochondria (shown in grey in the figure), even in the presence of oxygen in the surrounding environment as in the bladder lumen. In alcoholic fermentation, pyruvate is decarboxylated into acetaldehyde by pyruvate decarboxylase ①, and acetaldehyde is further metabolized to ethanol by alcohol dehydrogenase ②. TCA, tricarboxylic acid

Alcoholic fermentation is a metabolic process located downstream of glycolysis. Alcohol fermentation is required to maintain the upstream glycolysis steps by replenishing NAD^+^ anaerobically (Fig. 1) [10]. Most yeast species turn to glycolysis/alcoholic fermentation only when adequate oxygen is not available in their surroundings for energy production. However, some yeast species turn to alcoholic fermentation in response to excessive glucose in the environment, even in the presence of oxygen (called the Crabtree effect) [11]. The Crabtree effect is presumed to provide a survival and evolutional advantage for these yeast species over other microorganisms in a sugar-rich environment (e.g., ripened fruit) by suppressing the growth of other competing microorganisms with “toxic” ethanol. These yeasts are called Crabtree-positive [10, 12].

The bladder lumen contains oxygen (4–40 mmHg [13]), which allows only Crabtree-positive yeast to turn on glycolysis/alcoholic fermentation in response to excessive glucose in the bladder lumen (or hyperglycosuria due to poorly controlled diabetes in this case).

Among *Candida* species known to cause funguria [7], *C. glabrata* is Crabtree-positive, whereas other *Candida* species causing funguria (*C. albicans*, *C. tropicalis*) are all Crabtree-negative [10, 14, 15].

*C. glabrata* is phylogenetically much closer to *Saccharomyces cerevisiae* (*S. cerevisiae*), another Crabtree-positive fermenting yeast also known as “brewer’s yeast” or “baker’s yeast,” than to *C. albicans*. *C. glabrata* is normal human microflora that colonizes the surface of the mouth, esophagus, intestine, and vagina, but it can also cause fungemia in immunocompromised individuals. *C. glabrata* is the second most common cause of funguria after *C. albicans* [7, 14].

*S. cerevisiae* can also colonize the surface of the oral cavity, respiratory tract, gastrointestinal tract, and urogenital tract as occasional human microflora [16, 17]. *S. cerevisiae* has been regarded as a rare opportunistic pathogen; however, a recent report shows that urinary *S. cerevisiae* is associated with symptom flares of interstitial cystitis/bladder pain syndrome [18].

Based on the microbiological discussion, *C. glabrata* should be the primary yeast species that causes BFS, but *S. cerevisiae* can be another causative yeast of BFS. These predictions fit with the previous literature, including our case report [2]. In a previous forensic case report, *C. glabrata* was also identified as the causative yeast for fermentation in the urine specimen [4], and the authors suspected a pathophysiology equivalent to BFS.

### Physiological characteristics of the urinary bladder

**Fig. 2:**
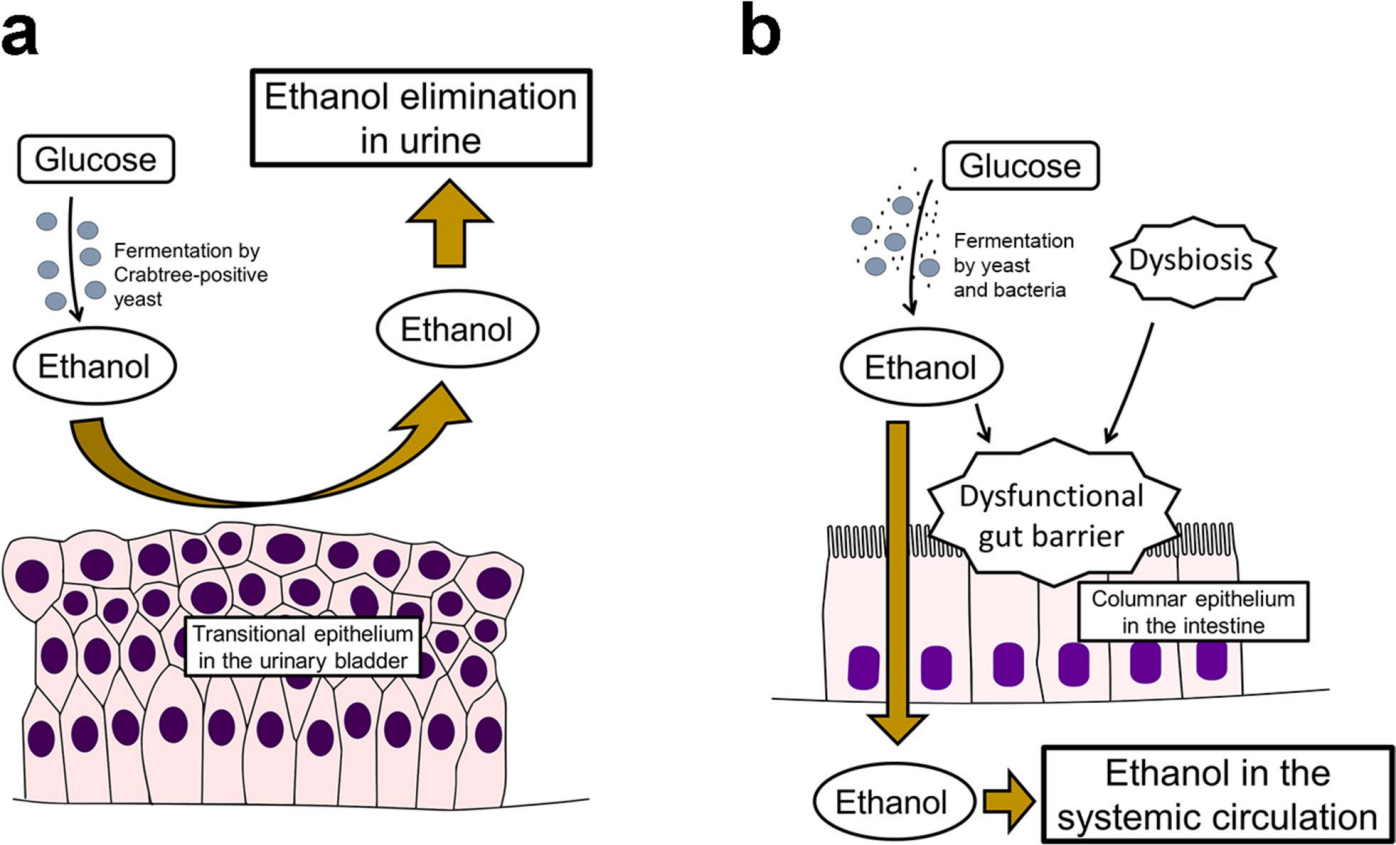
Pathophysiology of bladder fermentation syndrome and gut fermentation syndrome. **a** In bladder fermentation syndrome, ethanol produced through alcoholic fermentation by Crabtree-positive yeast is urinary-eliminated without getting absorbed into systemic circulation because the transitional epithelium in the urinary bladder serves as a barrier to ethanol. **b** In gut fermentation syndrome, ethanol produced through alcoholic fermentation by yeast and/or bacteria is absorbed into systemic circulation through the columnar epithelium in the intestine, causing alcohol intoxication. A dysfunctional gut barrier caused by dysbiosis and ethanol may also be involved in its pathogenesis

One crucial clinical characteristic of BFS patients is the lack of signs and symptoms of alcohol intoxication [2]. In BFS or bladder fermentation syndrome, alcohol in the bladder is not absorbed into the systemic circulation, leaving the patient sober. This property is attributed to the structure of the bladder wall. Histologically, the bladder lumen is covered by transitional epithelia, which function as barrier epithelia with extremely low permeability for water and small molecules such as ethanol (Fig. 2) [19, 20].

## Diagnostic tests of BFS

**Fig. 3:**
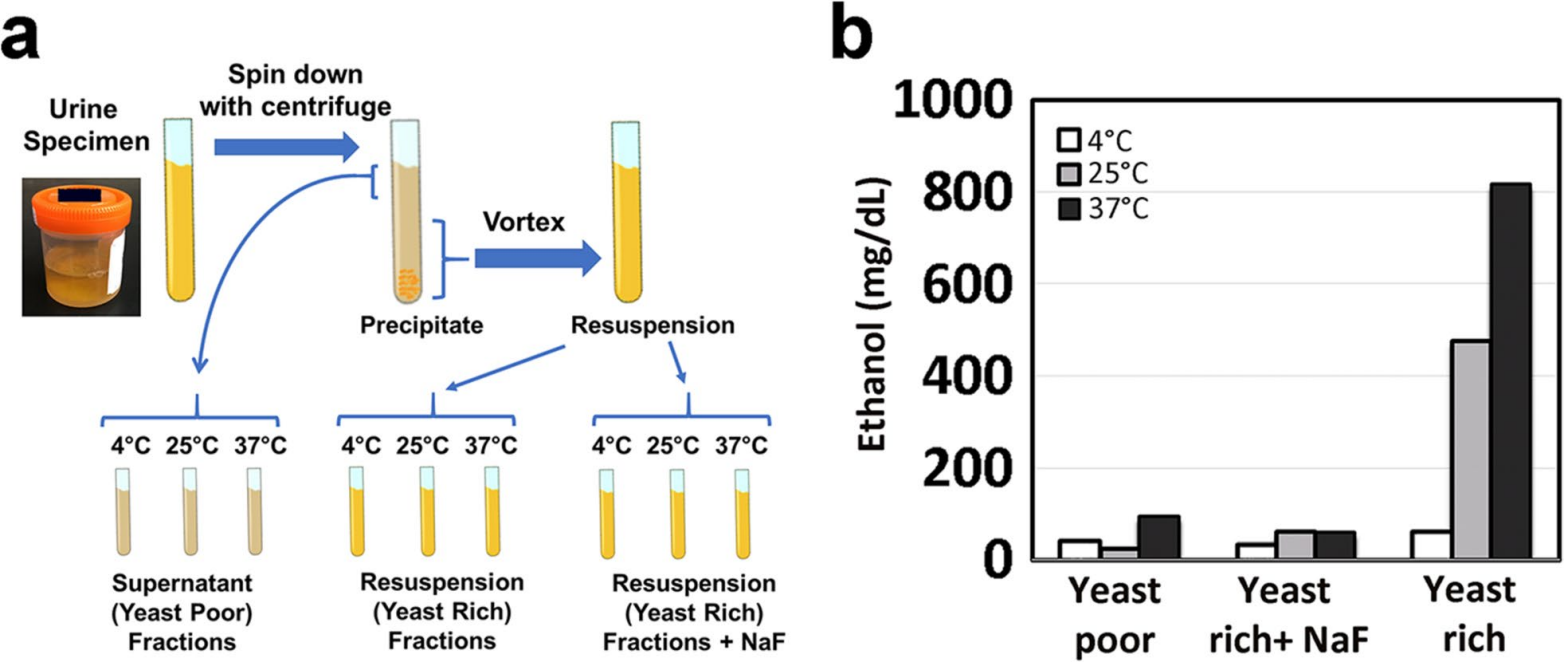
Ethanol production by fermenting yeast in bladder fermentation syndrome. **a** Outline of the experiment. In this experiment, the freshly voided urine sample was immediately transported to the laboratory on ice. The specimen was centrifuged into yeast-poor and yeast-rich fractions for incubation at three temperatures (4 °C, 25 °C, and 37 °C) for 24 h. The yeast-rich fraction was also incubated in the presence of 1% sodium fluoride, a fermentation inhibitor. The ethanol level was determined using headspace gas chromatography. **b** The ethanol levels in the urine sample after 24 h incubation. The ethanol level increased from 9.5 mmol/L (44 mg/dL) (baseline before incubation) to 103.3 mmol/L (476 mg/dL) and 177.1 mmol/L (816 mg/dL) after 24 h incubation at 25 °C and 37 °C, respectively. In contrast, minimal ethanol production was observed in the yeast-poor fraction and sodium fluoride and 4 °C conditions. No ethanol production was observed after 24 h incubation at 37 °C in the negative control urine (data not shown). The figure and legend reproduced with permission from Authors [2]. Urinary Auto-brewery Syndrome: A Case Report. Annals of Internal Medicine. 2020; 172: pp.702–704. https://www.acpjournals.org/doi/10.7326/L19-0661 ©American College of Physicians

**Fig. 4:**
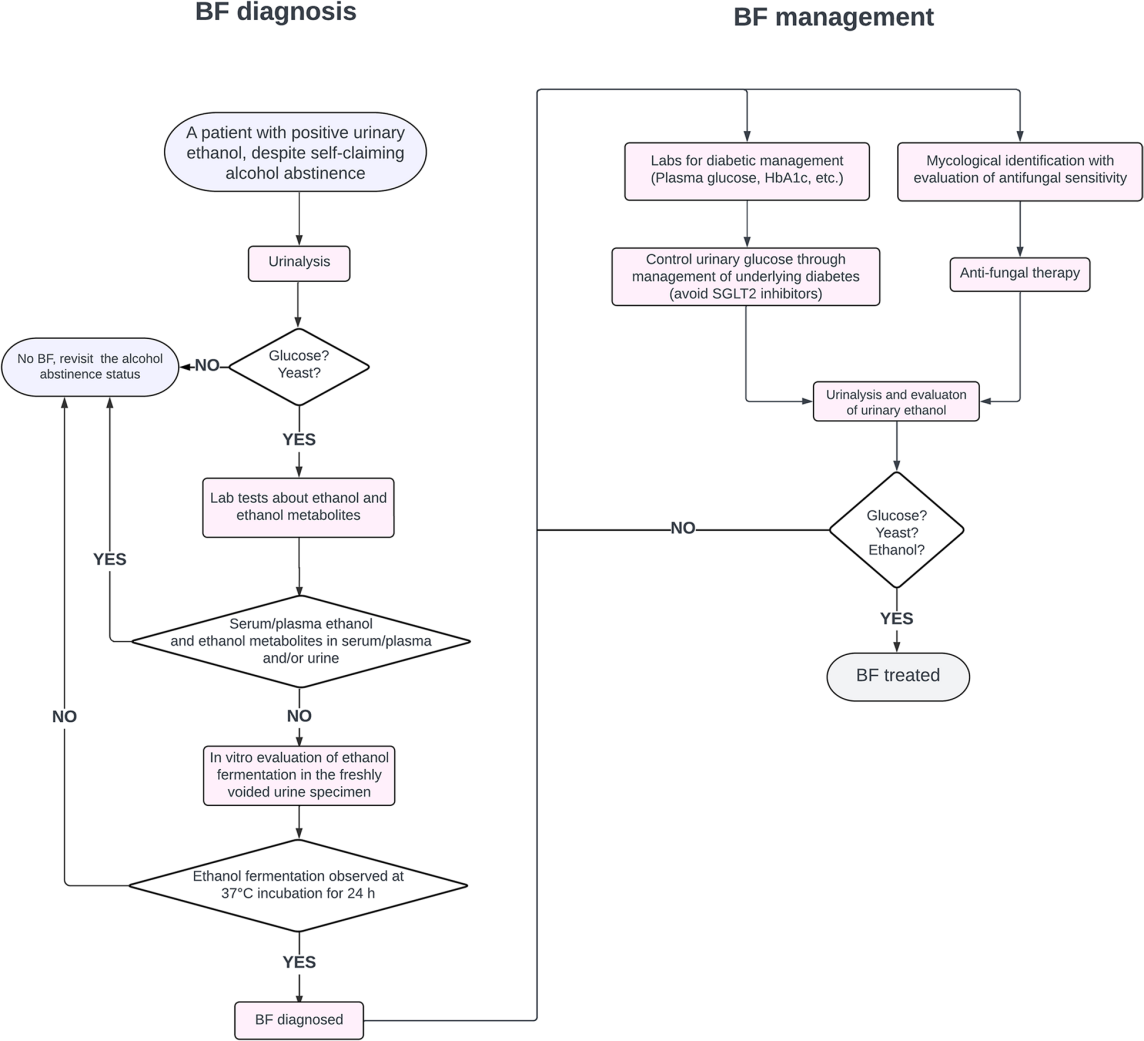
A flowchart of the proposed diagnostic testing and management of bladder fermentation syndrome. Please refer to the main text for detailed explanations. BFS, bladder fermentation syndrome

Based on the discussion of BFS pathophysiology, hyperglycosuria and colonization by Crabtree-positive yeast in the bladder are prerequisite conditions for BFS; both can be suggested by urinalysis. Laboratory identification of yeast species and antifungal susceptibility should be helpful for BFS diagnosis and management because the causative yeast should likely be *C. glabrata* or possibly *S. cerevisiae*. Ethanol metabolites (e.g., ethyl glucuronide or ethyl sulfate), created through the hepatic metabolism of ethanol, should be negative in BFS cases, as should phosphatidylethanol, another alcohol biomarker produced by a combination of ethanol and blood components [21] (Table 1). These test results are consistent with BFS, but a further specific experiment or a test battery was used to establish the BFS diagnosis (Fig. 3) [2], even though the entire investigation may be laborious and impractical for clinical laboratories. With the lab test results consistent with BFS (e.g., presence of glucose and Crabtree-positive yeast such as *C. glabrata* and negative ethanol metabolites in urine), the authors think that a simplified experiment to show additional ethanol production in the freshly voided urine in vitro at 37 °C incubation might suffice to establish the BFS diagnosis (Fig. 4).

**Table 1:** Key laboratory findings of bladder fermentation syndrome

Urinalysis	
**Biochemistry**	
Ethanol (serum/plasma)	Negative
Ethanol (urine)	Positive
Ethanol metabolites^a^	Negative
**Yeast identification**	
*Candida glabrata*	
(*Saccharomyces cerevisiae*)	

^a^Ethanol metabolites include ethyl glucuronide and ethyl sulfate in urine and phosphatidylethanol in serum/plasma

## Treatment of BFS

Treatment options for BFS are twofold: adequate control of hyperglycosuria (or underlying diabetes) and possible eradication of fermenting yeast in the bladder. Urinary glucose needs to be lowered to suppress fermentation. Diabetes itself is a risk factor for funguria; thus, adequate control of hyperglycosuria should also reduce the risk of yeast colonization in the bladder [7]. Regarding antidiabetic agents, sodium-glucose cotransporter 2 (SGLT2) inhibitors such as dapagliflozin inhibit SGLT2, which is responsible for glucose reabsorption in the glomerular filtrate by the renal tubules [22]. Thus, SGLT2 inhibitors might aggravate BFS by increasing urinary glucose excretion (Fig. 4).

Antifungal therapy might also be required to eliminate colonizing yeast in the bladder. However, both *C. glabrata* and *S. cerevisiae* produce biofilms, conferring resistance to azole-based antifungal drugs such as fluconazole [7, 14, 23]. Thus, antifungal therapy can be challenging, especially without reasonable control of diabetes (Fig. 4).

## Significance of BFS

### Alcohol abstinence evaluation

Alcohol abstinence monitoring is utilized in many clinical and legal situations, including alcohol addiction treatment programs, Driving Under the Influence (DUI) programs, driver’s license probation, and liver transplant evaluations. Self-reporting of alcohol use is generally not as reliable in certain situations, such as court-mandated treatment programs or being evaluated for a liver transplant, in which the discovery of alcohol use could lead to negative consequences [24].

Several laboratory tests have been proposed for abstinence monitoring. Ethanol itself is widely used as a biomarker of previous alcohol ingestion, but the narrow detection window of ethanol, usually hours, limits its utility as a biomarker for abstinence monitoring. Ethanol metabolites (e.g., ethyl glucuronide) are commonly used for abstinence monitoring due to their longer detection windows of previous alcohol ingestion [21].

Laboratory tests play a crucial role in establishing abstinence. Despite a clear indication for confirmatory toxicology monitoring, a standardized battery of abstinence biomarkers is not universally used in clinical practice. Furthermore, the interpretation of toxicology test results is not always straightforward [25]. The BFS can potentially mislead clinicians into a false assumption of alcohol consumption, resulting in the disqualification of an abstinent liver transplant candidate for transplant waitlist enrolment, as we reported before [2].

It is critically important for clinicians and laboratorians to know the limitations of these tests for reliable abstinence evaluation and to investigate any conflicting lab results. In addition to the awareness of potential exposure to ethanol through mouthwash and hand sanitizer, recognition of BFS and its prerequisite condition (e.g., uncontrolled diabetes with hyperglycosuria) should help clinicians conduct alcohol abstinence monitoring accurately.

### Forensic

As mentioned, there have been forensic cases in which BFS might have played a role. In the self-explanatory report entitled “Misleading results of ethanol analysis in urine specimens from rape victims suffering from diabetes,” two separate patients who adamantly denied alcohol use before sexual assault were reported to have high urine ethanol values after the incident (between 17.8 and 22.1 mmol/L (82 and 102 mg/dL)) in the background of poorly controlled diabetes with hyperglycosuria. The labs incorrectly suggested ethanol intoxication of the victims when the crime was committed. This short report recommends standardized testing for glucose in the urine and adding fermentation inhibitors such as sodium or potassium fluoride to the patient’s urine samples before specimen processing and measuring ethanol levels [5]. Presumed BFS has also been reported in post-mortem studies in forensic journals, the common denominator in all of these cases being patients with poorly controlled diabetes [4].

## Comparison between GFS and BFS

### Critical differences between GFS and BFS

If we contrast GFS with BFS, one of the key differences is the clinical manifestation of alcohol toxicity, as discussed earlier. A BFS patient, even with substantial ethanol in the bladder, would not have alcohol in their bloodstream and would not manifest symptoms of intoxication; thus, BFS patients can be asymptomatic and go unnoticed. In contrast, a GFS patient can develop alcohol intoxication spontaneously even without any alcohol ingestion, potentially resulting in adverse legal consequences such as DUI [26–31]. They may fail abstinence monitoring. The GFS patients may be symptomatic and thus seek medical treatment.

Both BFS and GFS are triggered by deranged microbiota within the hollow organs, leading to ethanol fermentation due to the overgrowth of fermenting yeast within the bladder and gut. To our knowledge, BFS appears to happen exclusively in the case of poorly controlled diabetes and resulting hyperglycosuria [2]. BFS should be caused by Crabtree-positive yeast, such as *C. glabrata*. On the other hand, GFS cases have typically been reported in patients with underlying medical conditions causing gut dysbioses, such as Crohn’s disease with strictures, short gut syndrome after surgery, or previous antibiotic use [27, 32–35]. A significant amount of ethanol is produced within the gut lumen, especially after consuming carbohydrate-rich food, causing alcohol intoxication in GFS patients [26, 27, 36]. The causative agents in most GFS cases are fermenting yeast, but high-alcohol-producing *Klebsiella pneumoniae (K. pneumoniae*) was recently identified as a causative microorganism of antifungal-resistant GFS [37, 38]. The partial pressure of oxygen within the gut lumen is less than 1 mm Hg [39], much lower than that of the bladder lumen. This near-anoxic environment should allow Crabtree-positive yeast (e.g., *C. glabrata, S. cerevisiae*), Crabtree-negative yeast (e.g., *C. albicans*), and facultative anaerobes (e.g., *K. pneumoniae*) to perform anaerobic fermentation within the gut lumen [27, 35–37, 40–42].

### Gut microbiota including mycobiota

Bacteria are dominant microorganisms in the gut microbiota. Nevertheless, the intestinal lumen of healthy human subjects also harbors fungal microbiota or mycobiota, with *Candida* spp. as the most dominant one [43]. The composition of mycobiota within the gut lumen is subject to various gut environmental factors [44]. One such factor is the bacterial microbiota in the gut lumen. The mycobiota population represents only a minor fraction of the entire gut microbiota in healthy subjects [45], but the gut mycobiota fraction increases significantly after antibiotic treatment [46]. This is consistent with previous reports indicating previous antibiotic treatments as a possible priming event of GFS [27, 32, 34].

The host diet is another gut environmental factor affecting the gut microbiota. For example, a carbohydrate-rich diet, a key precipitating factor for ethanol fermentation in the gut [26, 47, 48], is positively associated with the abundance of *Candida* spp. in gut mycobiota [49]. Ethanol is another factor causing dysbiosis with reduced fungal diversity and overgrowth of *C. albicans* [50, 51].

The gut microbiota uses glycolysis/alcoholic fermentation for ATP production. Even the serum/plasma specimens from sober subjects contain endogenous ethanol, although in trace amounts (0.14 mmol/L (0.66 mg/dL) on average) for healthy subjects derived from the intestinal microbiota [52]. The levels of endogenous ethanol are even higher in subjects with diabetes (1.05 mmol/L (4.85 mg/dL) on average), liver cirrhosis (3.45 mg/dL (0.75 mmol/L) on average), and both conditions (2.36 mmol/L (10.88 mg/dL) on average) [53]. Similarly, the ethanol levels in the portal vein blood from metabolic dysfunction-associated steatotic liver disease (MASLD) and MASH subjects are much higher (8.0 mmol/L (36.9 mg/dL) and 21.0 mmol/L (96.8 mg/dL) on average, respectively) than those in the healthy controls (2.1 mmol/L (9.7 mg/dL)), indicating the potential role of endogenous ethanol in the pathogenesis of MASLD and MASH [54]. Please note that the ethanol levels in portal blood are 186 (interquartile range, 17–516) times higher than those in peripheral blood in this study, indicating that most of the endogenous ethanol is eliminated through the first-pass effect by the liver, leaving the endogenous ethanol of MASLD and MASH patients in the peripheral blood likely below the cut-off level used in the clinical laboratories. However, ethanol fermentation in the intestine is significant at the pathological level and causes alcohol intoxication in GFS patients.

### GFS and fungal-type dysbiosis of the small intestine

Unlike BFS, which was not recognized until recently [2], clinical conditions similar to GFS were described in 1906 [55]. This syndrome was reported to display diverse clinical symptoms, including irritable bowel (e.g., diarrhea, abdominal pain), nutritional deficiency (e.g., vitamin B, zinc), psychoneurological symptoms (e.g., fatigue, depression), and respiratory catarrhal. It was responsive to antifungal drugs (e.g., nystatin) and diet modification with a reduction in yeast and fermentable carbohydrates. Based on the clinical features, this syndrome was proposed as part of fungal-type dysbiosis of the small intestine in the late 1990s, but no firm evidence of *Candida* involvement was obtained back then [56–58]. In GFS, clinical manifestations secondary to alcohol intoxication are more characteristic and prominent than so-called fungal-type dysbiosis. Nevertheless, GFS patients also display other symptoms, including irritable bowel, chronic fatigue, depression, non-food allergies, and general poor health [26, 27, 48, 59, 60], similar to fungal-type dysbiosis. Overall clinical features appear to overlap between fungal-type dysbiosis of the small intestine and GFS, but the latter displays more prominent alcohol intoxication.

### Dysfunctional gut barrier function and ethanol

As discussed earlier, the bladder lumen is protected by transitional epithelia impervious to small molecules including ethanol [19]. In contrast, gut epithelia are more permeable to ethanol. Additionally, ethanol causes dysfunctional gut barrier function or increased intestinal permeability by direct damaging/toxic effects on epithelial mucosa and ethanol-induced dysbiosis [61, 62]. Increased intestinal permeability is also reported in fungal-type dysbiosis of the small intestine [56].

Dysfunctional gut barrier function is also associated with irritable bowel syndrome (IBS), impaired nutrient absorption (e.g., vitamin B, zinc), food allergy, depression, and chronic fatigue [44, 63–67]. These symptoms related to dysfunctional gut barrier function largely overlap with those found in fungal-type dysbiosis and GFS patients [26, 47, 60]. Overall, dysfunctional gut barrier function is postulated to be another underlying pathogenesis of fungal-type dysbiosis and GFS, in addition to dysbiosis (Fig. 2).

### Diagnostic tests and treatment of GFS

In contrast to BFS, diagnostic testing and treatment for GFS have been proposed. The critical diagnostic test of GFS is a carbohydrate challenge test, in which blood or breath alcohol levels are monitored before and after oral glucose ingestion by the patient after overnight fasting. A concise protocol with ethanol monitoring 1 h after 5-g oral glucose ingestion was proposed in an earlier article [68]. In contrast, Malik et al. proposed a more comprehensive protocol of the carbohydrate challenge test with serial ethanol monitoring up to 24 h after oral ingestion of 200-g glucose. These authors also proposed conducting upper and lower endoscopies to harvest gastrointestinal secretions for microbiological study [27]. These comprehensive diagnostic tests and procedures will enable the clinical team to diagnose a GFS case confidently. However, the apparent under-recognition and poor acceptance of GFS among the medical community and uncertain health insurance coverage of these tests may deter clinicians from using these comprehensive diagnostic tests at once [69].

Similar to BFS, GFS is treated by modifying the underlying conditions and eradicating the fermenting microorganisms with antifungals and/or antibiotics. Carbohydrate-reduced diets and probiotics should also be supplemental to these regimens [26, 27, 37, 38]. One recent case report showed successful treatment of antifungal-resistant GFS by single fecal microbiota transplantation (FMT) [42]. In this case report, extensive antifungal regimens with fluconazole, nystatin, or amphotericin B were unsuccessful, even though *C. glabrata* was identified in a fecal culture. It is unclear if *C. glabrata* was the sole causative microorganism for GFS or if fermenting bacteria (e.g., high-alcohol-producing *K. pneumoniae*) were also involved in developing GFS in this patient. Nevertheless, this report indicates the critical involvement of gut dysbiosis in GFS pathogenesis and fecal microbiota transplantation as a promising therapeutic option for GFS.

### Prognosis of GFS and BFS

The prognosis and direct medical impact of BFS on patient health are unknown. Despite meta-analyses of epidemiological studies showing no significant association between bladder cancer and alcohol consumption [70, 71], a weak association was observed between alcohol consumption and the risk of bladder cancer in male subjects who consumed spirits or liquor, according to a dose–response meta-analysis [71]. This association is presumably due to acetaldehyde, which is suspected to act as a carcinogen for bladder cancer [72, 73]. Acetaldehyde is an intermediate metabolite of ethanol fermentation (Fig. 1), produced and released by *C. glabrata* from glucose within the bladder as well [74]. Therefore, the risk of bladder cancer development in untreated BFS patients may not be negligible over an extended period.

In contrast, recurrent acute ethanol intoxication without drinking or spontaneous inebriation occurs in untreated GFS patients [26–28, 30, 31]. Untreated GFS is also speculated to cause chronic ethanol intoxication in various organs, such as fatty liver disease. Recent reports show the critical roles of fermenting bacteria and yeast in the gut in the pathogenesis of MASLD and MASH [37, 54, 75], further supporting this speculation.

## Terminology

We proposed the term “bladder fermentation syndrome (BFS)” and “urinary auto-brewery syndrome” as an analogy to the previously known condition “gut fermentation syndrome (GFS)” or “auto-brewery syndrome” [2]. It can be argued that the terms “bladder fermentation syndrome (BFS)” and “urinary auto-brewery syndrome” are misnomers, since the term “syndrome” refers to a set of association symptoms, but the clinical symptom of BFS would only be urine scent. Nevertheless, BFS shares an underlying mechanism with the previously described, but rare GFS, and a part of the manuscript is dedicated to a comparison of the two conditions. Accordingly, we believe that readers and experts in the field will find the term “bladder fermentation syndrome (BFS)” or “urinary auto-brewery syndrome” more tangible.

The term “auto-brewery syndrome” was first introduced in an English peer-reviewed article in 1976 [41] and has been primarily used to describe GFS and its related conditions. According to Merriam-Webster, the word “brewery” means “a place where beer is produced.” Obviously, the gut and bladder are not places where beer is produced, and the term “auto-brewery syndrome” may be pejorative and stigmatizing to patients suffering from these conditions. Thus, we use the terms “gut fermentation syndrome (GFS)” and “bladder fermentation syndrome (BFS)” primarily in this manuscript.

At the time of preparing the manuscript, there is no consensus on the terminology used to describe these conditions. The underlying process in these conditions is the endogenous production of ethanol by fermenting microorganisms, which takes place more widely within the body than previously believed. The enhanced fermentation of ethanol can result in serious health consequences for patients. Enhanced ethanol fermentation in the gut can cause MASLD and MASH [54] or GFS with spontaneous alcohol intoxication if the endogenous ethanol generated in the gut is too high to be eliminated through the first-pass effect by the liver. Similarly, enhanced ethanol fermentation in the bladder cause BFS. For a more accurate description of these medical conditions, it would be more appropriate to use the terms “ethanol fermentation syndrome” or “elevated endogenous ethanol production” and its subclassifications.

## Conclusions

In this article, we reviewed the pathophysiology, clinical features, and key diagnostic tests of BFS with a comparative discussion with GFS. The under-recognition of these rare medical conditions can mislead medical professionals in the interpretation of abstinence monitoring due to repetitive positive results of urinary ethanol for BFS or serum/plasma ethanol for GFS, potentially precluding patients from accessing medical care including transplantation, as exemplified by our BFS case [2].

The paucity of scientific knowledge about fermenting yeast and bacteria in the body is one major reason for the under-recognition of these medical conditions. Future investigations will further elucidate the pathological roles of fermenting microorganisms not only in MASLD and MASH but also in GFS and BFS, hopefully raising awareness of these rare conditions in the medical communities. The use of new terminologies to reframe these medical conditions may also lead to a greater level of awareness among medical professionals.

## Data Availability

Not applicable.

## Abbreviations

BFS: Bladder fermentation syndrome
DUI: Driving under the influence
FMT: Fecal microbiota transplantation
GFS: Gut fermentation syndrome
MASH: Metabolic dysfunction-associated steatohepatitis
MASLD: Metabolic dysfunction-associated steatotic liver disease
NASH: Non-alcoholic steatohepatitis
SGLT2: Sodium-glucose cotransporter 2
